# 3D Printed Orthopaedic External Fixation Devices: A Systematic Review

**DOI:** 10.1186/s41205-023-00180-0

**Published:** 2023-06-07

**Authors:** Hunter A. O’Connor, Luke W. Adams, Lisa N. MacFadden, Nathan Wm. Skelley

**Affiliations:** 1 University of South Dakota Sanford School of Medicine, Sioux Falls, SD 57104 USA; 2 Sanford Orthopedics and Sports Medicine, 1210 W. 18th St, Sioux Falls, SD 57104 USA

**Keywords:** 3D printing, 3-dimensional printing, External fixation, Fracture, Stabilization, Orthopedics, Orthopaedics, Trauma

## Abstract

**Background:**

External fixators are complex, expensive orthopaedic devices used to stabilize high-energy and complex fractures of the extremities. Although the technology has advanced dramatically over the last several decades, the mechanical goals for fracture stabilization of these devices have remained unchanged. Three-dimensional (3D) printing technology has the potential to advance the practice and access to external fixation devices in orthopaedics. This publication aims to systematically review and synthesize the current literature on 3D printed external fixation devices for managing orthopaedic trauma fractures.

**Methods:**

The Preferred Reporting Items for Systematic Review and Meta-Analysis (PRISMA) protocols were followed for this manuscript with minor exceptions. PubMed, Embase, Cochrane Review, Google Scholar, and Scopus online databases were systematically searched. Two independent reviewers screened the search results based on predetermined inclusion and exclusion criteria related to 3D printing and external fixation of fractures.

**Results:**

Nine studies met the inclusion criteria. These included one mechanical testing study, two computational simulation studies, three feasibility studies, and three clinical case studies. Fixator designs and materials varied significantly between authors. Mechanical testing revealed similar strength to traditional metal external fixators. Across all clinical studies, five patients underwent definitive treatment with 3D printed external fixators. They all had satisfactory reduction and healing with no reported complications.

**Conclusions:**

The current literature on this topic is heterogeneous, with highly variable external fixator designs and testing techniques. A small and limited number of studies in the scientific literature have analyzed the use of 3D printing in this area of orthopaedic surgery. 3D printed external fixation design advancements have yielded promising results in several small clinical case studies. However, additional studies on a larger scale with standardized testing and reporting techniques are needed.

## Background

Three-dimensional (3D) printing has had a multifaceted impact on orthopaedic surgery. Its applications include wide-ranging uses such as customizable cutting guides, prosthetics, splints, sterile implants, anatomic models for surgical planning, patient education, and patient-specific instrumentation [1–5]. One application of 3D printing in orthopedics that has had growing interest in recent years is the use of 3D printing for the production of external fixation devices. External fixation is an integral technique in orthopedics with many indications including, but not limited to, temporary stabalization of fractures, limb lengthening, limb reconstruction, correction of non-union deformities, arthrodesis, and management of osteomyelitis [6].

**Fig. 1:**
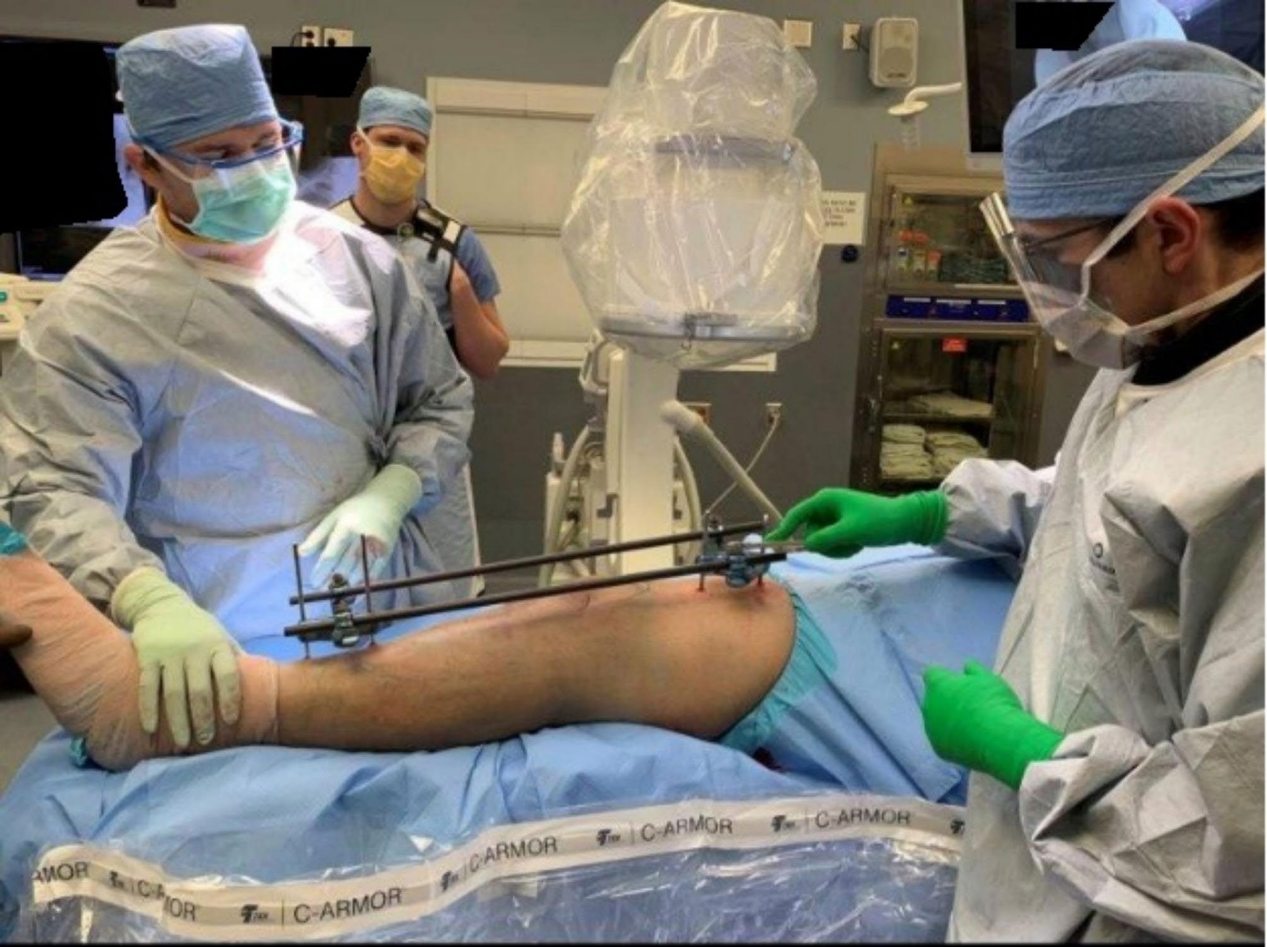
Orthopaedic surgeons use fluoroscopy to stabilize and span a comminuted proximal tibia fracture. Metal partially threaded half pins, frequently called Schanz screws, are placed far away from the zone of injury around the knee. Composite rods span the traumatized area, providing axial, bending, and rotation stability. They allow the swelling and soft tissues to recover before definitive surgical fracture fixation at a later date.

External fixation is the process of stabilizing a limb by using metal pins drilled into bone to attach external scaffolding or tensioning wires [6]. The pins are inserted percutaneously into the bone through small incisions in the skin. External fixators can be broadly classified based on the positioning of the pins and configuration of the scaffolding structures, which are referred to as frames. The major categories of external fixators are circular, unilateral, multiplaner, and hybrid designs. Circular fixators, such as the commonly used Ilizarov device, fully encircle the limb with a ring structure and attach at multiple points around the circumference of the bone. Unilateral fixators, such as the trauma type pin-to-bar fixators, are positioned on only one side of the limb (Fig. 1). Multiplaner devices have pins in multiple planes, such as the transverse and sagittal planes [6]. Finally, hybrid external fixators incorporate aspects of both circular and unilateral fixators. Pins are partially threaded metal implants that are drilled into the bone to secure the external fixator frames to the bone. They can be subcategorized into full pins and half pins (also called Schanz screws). Full pins pass completely through the limb and protrude out of the skin on both sides. Half-pins only penetrate one side of the limb and are inserted to a distance to obtain purchase in both cortices of the bone and no further [7].

One of the most common orthopedic indications for external fixation is to temporarily stabilize a fracture prior to definitive treatment with Open Reduction and Internal Fixation (ORIF). Significant soft tissue swelling and skin damage often prevents ORIF in the period immediately following injury. An external fixator can be used in the interim to hold the fracture in an approximated position while the soft tissues recover [6, 8]. For a minority of patients, external fixation must be used as the definitive treatment option [9]. A recent retrospective study of 341 fractures treated with external fixation at a level 1 trauma center reported that 8% of external fixators were used as definitive treatment [10]. Obtaining an accurate initial reduction with external fixation is imperative when external fixation is used as the definitive treatment, as inaccurate reduction can lead to complications such as malunion or delayed bone healing [9, 11]. However, for some fractures, it can be difficult to achieve the same level of reduction accuracy as ORIF with current external fixation techniques, because the surgeons cannot directly visualize and manipulate the fracture ends [9]. Current techniques also require significant radiation exposure to the patient and physician, as repeated x-rays are taken to ensure adequate fracture reduction.

Utilizing 3D printing to manufacture external fixators could address several critical shortcomings of current external fixation techniques. Most notably, new 3D printing technology in combination with 3D computer modeling of fractures has enabled the development of personalized 3D printed external fixation devices that improve fracture reduction accuracy and reduce the need for intraoperative x-rays [9, 12, 13]. 3D printing also has the potential to reduce costs associated with external fixation significantly. The external fixation devices that are currently commercially available are expensive [14–16]. One retrospective study at a level one trauma center in the United States reported that the average external fixator cost was $5,900 and the total yearly expenditure at the facility surpassed $650,000 [10]. The high cost of external fixators is especially problemeatic in low income areas such as developing countries. A lack of affordable options limits access to external fixation technology in these low income areas [14–16]. 3D printed external fixators would likely cost less to produce than current fixators because 3D printed materials are typically less expensive than materials currently used, such as steel or carbon fiber.

Incorporating 3D printing technology into external fixator production represents a significant opportunity for improvements in cost, simplicity, customization, and accuracy of external fixator constructs. The purpose of this manuscript is to systematically review and synthesize the current literature on 3D printed external fixation devices for managing orthopaedic trauma fractures.

## Methods

### Literature Search

We followed the Preferred Reporting Items for Systematic Review and Meta-Analysis (PRISMA) protocols with minor exceptions [17]. In February 2023, we searched the PubMed, Embase, Google Scholar, Scopus, and Cochrane Review computerized databases for records published from January 1950 to March 2023. The search terms included: “three dimensional printing,” “external fixation,” “fracture,” “orthopaedics,” and all associated synonyms. The full search strategies for each database are given in Table 1. Indexing terms and functions for Google Scholar differed from the other databases, so the authors employed a slightly different search strategy for this database. Publications identified in these searches were imported into Zotero bibliography software [18] to identify and exclude duplicate and retracted articles. Studies were included in the systematic review if they involved developing or testing external fixation devices for use in orthopaedic fracture management or stabilization with at least one 3D printed component. Studies were excluded if they did not involve 3D printing or external fixation devices, if they featured external fixation devices without 3D printed components, or if they examined external fixation devices in areas of medicine other than orthopaedics and for uses other than fracture management. Any language was accepted as long as the publication could be translated into English using Google translation software [19, 20]. Two authors (HAO and LWA) independently screened the articles based on title and abstract and selected those that met the inclusion and exclusion criteria. The full text of the selected articles was then reviewed to confirm that all criteria were met. The reference lists of these articles were then manually searched for other relevant articles. All authors agreed on the final list of articles following the full-text review process.

**Table 1:** Search strategies

Databases	Search Strategy
Embase, PubMed, and Cochran Review	(external fixation OR ex fix OR ex-fix OR external fixator) AND (three dimensional printing OR three-dimensional-printing OR 3d printing OR 3d printed OR 3d-printed OR 3 dimensional printing OR 3-dimensional-printing OR 3d-printing OR fused deposition modeling OR fdm OR additive manufacturing OR additive layer manufacturing) AND (orthopaedics OR orthopedics OR orthopaedic surgery OR orthopedic surgery OR fracture OR bone OR trauma)
Google Scholar	(external fixation OR ex-fix OR external fixator) AND (three dimensional printing OR 3d printing OR 3d printed OR fused deposition modeling OR additive manufacturing) AND (orthopedics OR orthopaedics OR orthopedic surgery OR fracture OR bone OR trauma)

### Classification and Review

Included papers were classified by country of origin, language, fracture location, and study type. Study types were defined to include (1) clinical case reports, a review of an individual case or case series (2 + patients) using a 3D printed external fixator device, (2) feasibility studies, that explored the feasibility of using 3D printed external fixators to manage fractures in cadaveric or model bones, (3) mechanical testing studies in which mechanical testing was conducted on 3D printed external fixation constructs and (4) computational simulation studies using finite element models to analyze and optimize the mechanical properties of 3D printed external fixator devices. Included papers were read by all authors and are described in detail below. In exception to the PRISMA protocols, we did not assess the risk of individual study bias or the strength of the overall body of evidence using a method such as GRADE. These items were not assessed due to the heterogeneity of studies and because this manuscript’s purpose of discovering all studies in this area of orthopaedic surgery did not depend on the quality of individual studies.

## Results

**Fig. 2:**
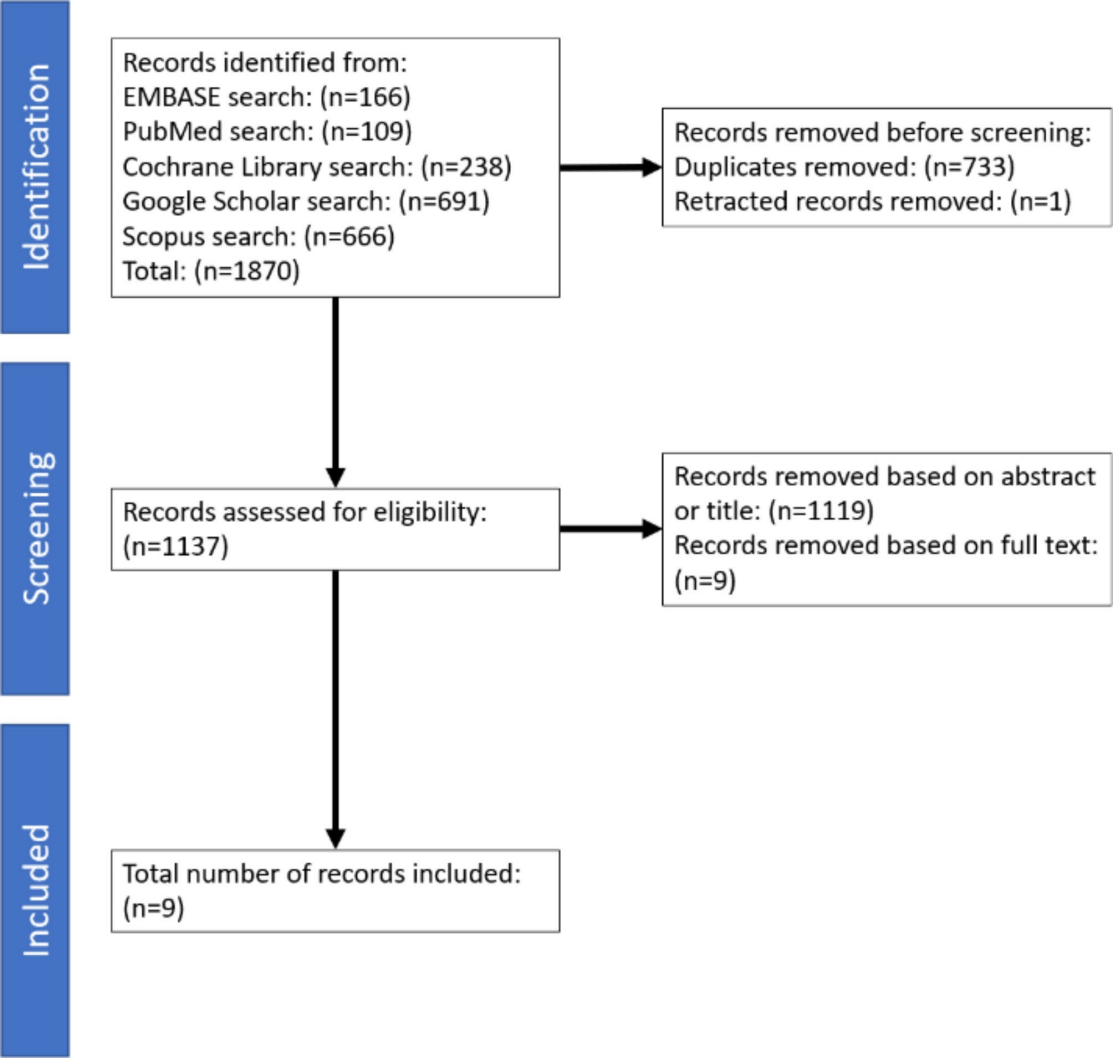
Systematic review: literature identification and screening process

The database search returned 109 articles from PubMed, 166 articles from Embase, 238 articles from Cochrane Review, 691 articles from Google Scholar, and 666 articles from Scopus for a total of 1,870 articles (Fig. 2). Of the 1,870 articles identified, 733 were duplicates, and one was retracted, resulting in a final list of 1,137 articles to review against the inclusion and exclusion criteria. After inclusion and exclusion criteria were applied, nine studies remained (Table 2) [9, 12–14, 21–25].

The nine studies included in this systematic review were published between 2015 and 2021 and were conducted globally. Five studies were conducted in China [9, 12, 13, 21, 24], one study was conducted in Germany [25], one in the United States [14], one in the United Kingdom [22], and one in Russia [23]. All articles were available in English, except for the study by Huang et al. [21] which was written in Chinese and was translated to English using Google translation software. Four studies focused on external fixator devices for managing tibial shaft fractures [9, 13, 21, 24], one study focused on distal radial epiphyseal fractures [23], one study focused on femur fractures, and one study evaluated both femur and tibia fractures [12]. Two studies did not focus on evaluating their devices for a specific fracture location [14, 22]. The included studies also covered a broad range of topics related to external fixation. Three studies evaluated clinical case outcomes with 3D printed external devices for fracture management [9, 13, 21] and three studies explored the feasibility of using 3D printed external fixators to manage fractures [12, 23, 25]. In addition, one study reported results from mechanical testing of external fixator constructs [14], and two studies used computational simulation to evaluate the design and optimization of external fixator devices [22, 24].

**Table 2:** Summary of included studies

Study	Country	Fracture Location	Study Type
Qiao et al. (2015) [12]	China	Tibia/Femur	Feasibility
Qiao et al. (2016) [13]	China	Tibia	Clinical Case Study
Huang et al. (2016) [21]	China	Tibia	Clinical Case Study
Omar et al. (2017) [25]	Germany	Femur	Feasibility
Golubev & Petrov (2019) [23]	Russia	Distal Radius	Feasibility
Landaeta et al. (2020) [14]	USA	NA	Mechanical Testing
Alqahtani et al. (2021) [22]	UK	NA	Computational Simulation
Li et al. (2021) [24]	China	Tibia	Computational Simulation
Wu et al. (2021) [9]	China	Tibia	Clinical Case Study

### Device Characteristics

The external fixation device characteristics are summarized in Table 3, including the fixator type, description of the device, 3D printed material used, and 3D printing technique. The studies were further classified into those that described fixators that are personalized to patients’ specific fracture characteristics and those that developed nonpersonalized fixators. Several different materials were used, including acrylonitrile butadiene styrene (ABS) in two studies [21, 23], nylon in two studies [9, 14], polylactic acid (PLA) in two studies [22, 24], photosensitive resin in two studies [12, 13], and polyjet photopolymer MED610 in one study [25]. Multiple different 3D printing techniques were also used, including fused deposition modeling in two studies [14, 21], vat photo-polymerization in two studies [12, 13], and material jetting in one study [25]. Three studies did not report a specific 3D printing technique [9, 22, 23]. Seven studies described novel personalized, patient specific devices that provide spontaneous fracture reduction during application [9, 12, 13, 21, 22, 24, 25]. In addition, two studies developed nonpersonalized 3D printed external fixation components to augment or replace components of external fixators that are currently available [14, 23]. The features and appearance of each device varied significantly between studies and are described in detail below.

**Table 3:** External Fixation Device Characteristics

Study	Fixator Type	Description	3D Printed Material	3D Printing Technique	Personalized?
Qiao et al. (2015) [12]	Circular	Q-fixator based on Ilizarov	Photosensitive resin	Vat Photo-polymerization	Yes
Qiao et al. (2016) [13]	Circular	Q-fixator based on Ilizarov	Photosensitive resin	Vat Photo-polymerization	Yes
Huang et al. (2016) [21]	Multiplaner	Triangular Bracket	ABS	Fused Deposition Modeling	Yes
Omar et al. (2017) [25]	Temporary Reduction Aid	Two Rectangular Blocks	Polyjet Photopolymer MED610	Material Jetting	Yes
Golubev & Petrov (2019) [23]	Unilateral	Pin to Bar with 3D Printed Hinge	ABS	Not Reported	No
Landaeta et al. (2020) [14]	Unilateral	Pin to Bar with 3D Printed Clamps	Nylon with Chopped Carbon Fiber	Fused Deposition Modeling	No
Wu et al. (2021) [9]	Circular	Limb Contouring Circumferential Shell	Reinforced Nylon	Not Reported	Yes
Li et al. (2021) [24]	Circular	Circumferential Shell of Removable Voronoi Shapes	PLA	Fused Deposition Modeling	Yes
Alqahtani et al. (2021) [22]	Circular	Limb Contouring Circumferential Shell	PLA	Not Reported	Yes

The device described by Qiao et al. is a variation of the classic circular Ilizarov device and is personalized to the specifics of each fracture [12, 13]. This device is constructed from photosensitive resin using vat photo-polymerization and involves two identical cylindrical structures that encase the limb. One cylinder is attached to the proximal bone fragment and one to the distal fragment. The cylinders are connected axially by four threaded rods and nuts. Each cylinder is made up of two semicircular modules that are connected with bolts during installation. The cylinders are attached to the bone with one full pin and two half pins each. This results in four points of contact for each cylinder and eight points total. This device is designed on a computer using 3D imaging of the patient’s fracture. The fracture reduction is simulated on a computer, and the device is designed to spontaneously achieve fracture reduction when the two cylinders are connected to each other axially during application. The axial compression on the fracture site can be adjusted by tightening the nuts on the axial threaded rods.

Huang et al. described a personalized multiplanar solid triangular bracket external fixator device constructed from ABS using fused deposition modeling [21]. The fixator is attached to the bone fragments with two half pins in the distal fracture fragment and two half pins in the proximal fracture fragment. The proximal pins are inserted perpendicular to the distal pins, with the proximal pins oriented in the transverse plane and the distal pins oriented in the sagittal plane of the tibia to increase the stability of the construct.

Wu et al. described a personalized circular external fixator device that consists of a single shell made out of reinforced nylon that encases the lower limb [9]. The form of the device follows the contour of the patient’s leg with a uniform space between the skin and the device along the entire structure. The device is connected to three half pins in the distal fragment and three half pins in the proximal segment. The final fixator construct results from the combination of 2–3 submodules that are connected during installation. During the design process performed in a computer, all structurally redundant materials were removed, thus optimizing the strength-to-weight ratio of the device, resulting in a lightweight cage-like final construct.

Alqahtani et al. developed a personalized circular external fixator with optimal strength-to-weight ratios [22]. Their design consisted of a porous shell made to fit around the circumference of the leg. Similar to Wu et al., the form of the device follows the contour of the patient’s leg with a uniform space between the skin and the device along the entire structure. Leveraging finite element analysis (FEA) and statistical analysis, they determined design parameters (including optimal thickness, design type, and fixator material) that maximized strength and stiffness and minimized weight for the construct. Their final design was 4 mm thick, topology optimized, and printed in PLA.

Landaeta et al. developed a nonpersonalized unilateral trauma type pin to bar external fixation design that resembles clinically available linear external fixation designs but with the addition of 3D printed connecting clamps [14]. The design involves standard 12 mm steel rods attached using 3D printed clamps to two half pins in the distal fracture fragment and two half pins in the proximal fragment. These clamps are printed from nylon reinforced with chopped carbon fiber using fused deposition modeling. The final clamp is constructed from two separate clamp components: one that connects to the rod and one that connects to the pin. These two clamp components are connected using a nut and bolt.

Omar et al. presented a personalized 3D printed fracture reduction aid that spontaneously reduces fractures prior to attaching a traditional unilateral external fixator [25]. The design consists of a pair of rectangular blocks that are merged together and slid onto two half pins distal to the fracture and two half pins proximal to the fragment. There are two canals on the surface of each block that align with the pins when the fracture is in a reduced position and act as a locking mechanism when the blocks are slid onto the pins. The blocks are printed in polyjet photopolymer MED610 using material jetting technology.

Li et al. presented a model for a 3D printed patient-specific circular external fixator with adjustable stiffness that expands on their previously described Q-fixator [24]. Their device consists of a block structure external fixator with a shell of randomly distributed Voronoi shapes ranging in size from 2 to 5 cm offset by 1 cm. The form of the device follows the contour of the patient’s leg with a uniform space between the skin and the device along the entire structure. A tubular structure connects adjacent Voronoi shapes with a diameter of 1 cm. The construct is composed of two anteroposterior main frame parts connected by four sets of studs and nuts. The fixator is connected to the bone with two full pins and four half pins fixed to the center of Voronoi shapes. Two actuators were also implemented to prevent pin loosening. The fixator was printed with PLA through fused deposition modeling. The stiffness of the proposed design can be adjusted after application by removing individual Voronoi shapes according to the stiffness tolerance of the healing callus during different stages of fracture healing. The adjustable stiffness enables an optimal healing environment at successive stages of fracture healing.

Golubev and Petrov developed a nonpersonalized unilateral hinged external fixator for distal radius epiphyseal fractures [23]. The multidirectional hinge (or “hub”) is 3D printed with ABS and consists of two separate blocks that articulate with one another on a circular surface, allowing for wrist movement in both the palmar-dorsal plane and the ulnar-radial plane. The hinge forms an articulating connection between two pin to rod standard external fixators that are secured to the proximal and distal bone fragments. The distal external fixation rod is attached to two half pins inserted transversely through two metacarpal bones. The proximal rod is attached to two half pins inserted transversely into the distal radius. The entire device sits medially along the radial side of the wrist. The hinge allows for early wrist joint mobility, which may improve functional outcomes [26].

### Design, Manufacturing, and Installation Process for Personalized External Fixators

The design, manufacturing, and installation process followed the same general steps for all seven personalized external fixators. [9, 12, 13, 21, 22, 24, 25]. First, pins were inserted into the proximal and distal bone fragments with x-ray guidance. A CT scan was then obtained with the pins in place. A temporary external fixator was placed in all three clinical studies while the 3D printed fixator was manufactured. The CT scan was used to render a computerized 3D representation of the fracture. Next, a fracture reduction simulation was conducted by manually manipulating the 3D images of the proximal and distal fracture fragments to identify the pin positions that reduced the fracture. In the case study by Huang et al. [21], the highly comminuted nature of the fracture necessitated that the contralateral limb be used as a template to determine appropriate fracture positioning during the computer simulated fracture reduction. A 3D model of the external fixator was then constructed with CAD software to coincide with the pin positions from the simulated reduction. The CT images of the soft tissue were used to adjust the size of the external fixator to fit appropriately around the limb. In the Qiao et al. study, this process was used to determine the diameter of the cylinders [13]. Wu et al., Alqahtani et al., and Li et al. used an expanded contour of the patient’s soft tissue to produce the initial shape of the fixator [9, 22, 24]. The fixators were then 3D printed and installed by aligning and connecting the mounting holes of the fixator with the pins, which required traction on the fracture. Analgesia or anesthesia was given to all patients during the procedure in clinical studies. Fracture reduction was spontaneously achieved during installation without the need for intraoperative x-rays. Postoperative x-rays were obtained to ensure proper reduction in all clinical studies. In contrast to the other studies, Omar et al. manufactured a temporary reduction aid rather than a definitive external fixator [25]. In this study, a definitive traditional unilateral rod to pin external fixator was attached to the pins after fracture reduction was achieved, and the reduction aid was subsequently removed.

### Mechanical Testing Results

Landaeta et al. was the only group to assess the mechanical properties of a 3D printed external fixator. [14]. Appropriate methods from the American Society for Testing and Materials (ASTM) standard F1541 were applied to quantify the mechanical properties of the construct. Axial compression, anterior-posterior (AP) bending, and medial-lateral (ML) bending were tested using a mechanical testing system, and load and displacement were measured. Rigidity, safe load, and yield load were computed from the load-displacement data. The clamps’ diameter, width, height, and length were measured before and after autoclaving and shrunk by 2.6%, 0.2%, 1.7%, and 0.3%, respectively, with all dimensions remaining within +/- 0.125 mm of the original dimensions. The average safe load from axial compression, defined as the load required to produce 1 mm of axial displacement, was 177.14 N. The average yield load, defined as the load required to cause deviation from the initial linear trend of the load vs. displacement curve, was 122.92 N. The average axial rigidity of their external fixator was 246.12 N/mm, comparable to the rigidity of two external fixators currently in clinical use: Stryker’s stacked half-frames Hoffman external fixation system (258 N/mm) and Synthes’ AO stacked half frames Hoffman external fixation system (150 N/mm) [27]. The average AP and ML bending rigidities of the external fixator in this study were 35.98 N/mm and 39.6 N/mm, respectively. These values are higher than the reported ML and AP bending rigidity values for Stryker’s Hoffman 3 external fixator, which range from 12 to 39 N/mm depending on the configuration [18]. The production cost of their external fixator was less than $150, which was noted to be significantly less than devices currently in use which range in price from $3556 to $20,486.

### Feasibility Study Results

Qiao et al. reduced saw cut fractures in a foam femur, cadaveric femur, and cadaveric tibia model using their Q-fixator 3D printed external fixator design [12]. A binocular 3D measurement system that analyzed the location of several markers placed on the proximal and distal bone fragments prior to cutting the bones was used to determine the rotational, angulation, and lateral displacement errors after reduction. Satisfactory reduction was achieved in all three trials with average rotational, angulation, and lateral displacement error values of 1.21 degrees +/-0.24, 1.84 degrees +/-0.28, and 2.22 mm +/-0.62, respectively. Omar et al. reported an effective reduction of an animal femur using their 3D printed fracture reduction aid [25]. However, they did not report specific displacement measures after reduction. Finally, Golubev & Petrov tested the range of motion of a cadaver wrist without fracture when attached to their external fixator hub/hinge device [23]. They determined that the wrist range of motion with the external fixator attached was not significantly different than before fixator attachment.

### Computational Simulation Results

Alqahtani et al. performed finite element and statistical analyses to determine the optimal porous element design, construct thickness, and material for their 3D printed external fixator [22]. First, they investigated how changing the number and size of porous elements affected construct strength and stiffness. The configuration with the smallest diameter and the largest number of porous elements performed best, so this configuration was used for the remaining analyses. Next, the authors examined how changes in thickness, type of 3D printed material used, and the shape of porous elements (hexagon, circle, or topology optimized design) affected the strength and stiffness of the constructs. They found that each design parameter had a statistically significant influence on strength and stiffness. Specifically, the fixator thickness had a positive relationship with strength and stiffness. Printing with PLA resulted in the highest strength and stiffness, followed by ABS. Nylon had the lowest strength and stiffness. The topology optimized design had higher strength and stiffness than the devices with hexagonal or circular porous elements. Finally, Minitab statistical software was used to optimize weight and maximize the strength and stiffness of the external fixator. The optimal fixator construct had a thickness of 4 mm, was printed in PLA, and utilized topology optimization to determine the shape and size of porous elements.

Li et al. [24] used CT scans of one of the tibial fracture cases from their group’s previous case series [13] to test their novel adjustable stiffness external fixator in the 3D modeling software Rhino. They performed a finite element simulation on the fixator and fracture, which reinforced the inverse relationship between the stress distribution at the fracture gap and stress on the external fixator. The authors designed the device such that the stiffness of the external fixator could be adjusted by dismantling the construct in a sequence of eight steps with the following incremental decreases in stiffness with each step: 1%, 1.11%, 1.11%, 6.11%, 2.4%, 7.7%, 2.9%, and 0.14%. Based on previous studies examining the amount of strain tolerated by bone at different stages of healing, the stiffness adjustment tolerances were set at < 2% for the primary stage of bone healing and 2–10% for the secondary stage of bone healing. The first three steps of the dismantling sequence, 1%, 1.11%, and 1.11%, caused a stiffness decrease that was < 2% and could therefore be used in the primary stage of bone healing. Steps four through seven of the dismantling sequence, 6.11%, 2.4%, 7.7%, and 2.9%, caused stiffness changes between 2 and 10% and, therefore, could be used during the secondary stage of bone healing.

### Clinical Case Study Results

Table 4 summarizes the clinical results of the five patients treated across all studies with 3D printed external fixation for tibial shaft fractures. One study included three patients [13] and the other two studies included one patient [9, 21]. One of the five patients had an open fracture requiring closure with sutures [13]. In the case study by Huang et al., a traditional unilateral pin to rod external fixation device was initially attempted. This resulted in extreme limb shortening and angulation, and led to the decision to utilize a 3D printed fixator [21]. The average patient age was 31, and four of the five patients were male. The time for the application of the fixator was shared in two of the three papers and averaged 9 min [13, 21]. No x-rays were required during the installation of the fixator in any of the studies, and every patient had satisfactory initial reduction after the application of the external fixator. Table 5 summarizes the postoperative fracture displacement measurements reported by each study. Comparing the results between studies is difficult as different displacement metrics were reported. The largest reported linear displacement in any direction was 4.5 mm, and the largest angular displacement was 4°. The patients in the study by Qiao et al. were allowed to start full weight-bearing four weeks after surgery, and all patients ambulated without pain [13]. The external fixator served as a definitive treatment for all cases. The average time of fixator application was 19 weeks and ranged from 12 to 25 weeks. No loss of reduction or complications were reported in any of the studies.

**Table 4:** Clinical results for each patient managed with 3D printed external fixation

Study	Fracture	AO Classification Type	Age (years)	Sex	Operative Reduction Time (min)	Satisfactory Reduction Achieved?	Application (weeks)	Loss of Reduction	Complications
Qiao et al. (2016) [13] Patient 1	Tibial Shaft	42A2	36	Male	9	Yes	20	No	None
Qiao et al. (2016) [13] Patient 2	Open Tibial Shaft	42B3	30	Male	8	Yes	22	No	None
Qiao et al. (2016) [13] Patient 3	Tibial Shaft	42B3	25	Male	9	Yes	25	No	None
Huang et al. (2016) [21]	Tibial Shaft and Fibula	42C3	18	Male	10	Yes	14	No	None
Wu et al. (2021) [9]	Tibial Shaft and Fibula	42C2	46	Female	Not Reported	Yes	12	No	None

**Table 5:** Postoperative fracture displacement

Study	Varus/Valgus Displacement (°)	Anteversion/ Retroversion (°)	Axial Displacement (mm)	Radial Displacement (mm)	Anterior Posterior Displacement (mm)	Lateral Displacement (mm)	Angular Displacement (°)
Qiao et al. (2016) [13] Patient 1	1.05	3.12	0.42	0.42	-	-	-
Qiao et al. (2016) [13] Patient 2	2.63	0.85	4.50	2.30	-	-	-
Qiao et al. (2016) [13] Patient 3	3.46	4.13	1.94	2.66	-	-	-
Huang et al. (2016) [21]	1.7	2	-	-	-	-	-
Wu et al. (2021) [9]	-	-	-	-	0.15	1.08	0

## Discussion

There is a paucity of studies on 3D printed external fixation devices in the scientific literature [9, 12–14, 21–25]. While the available research on 3D printed external fixators represents work done worldwide, the majority (5 out of 9) of publications in this review are from research groups in China. This systematic review demonstrates significant variability in design, utilization, and testing techniques among the existing studies. Different circular and unilateral 3D printed external fixation constructs have been developed and tested. These fixator devices were constructed from various 3D printed materials, including PLA, ABS, photosensitive resin, and nylon-based materials. Only one study tested the mechanical properties of a 3D printed external fixator [14]. This study tested a unilateral pin to rod fixator using 3D printed clamps, and found the fixator to have similar rigidity properties to metal external fixators currently in use. This study was also the only study that reported material costs, which were less than 5% of the cost of external fixators that are commercially available. Further mechanical testing is required to understand the structural capacity of circular external fixation devices and other fixators constructed with 3D printed material. Only a single study conducted an ex-vivo pilot of fracture reduction using a 3D printed external fixation device [12]. Sufficient fracture reduction in cadaver and foam models was achieved in this study using their novel Q-Fixator design. Based on the results of this study, the authors decided to move forward with clinical testing of their device [13]. Although positive results have been demonstrated in these early feasibility studies, additional data is required before large-scale clinical testing can be conducted.

Customizability is one of the advantages of 3D printing technology over prefabricated products. Most of the available studies on 3D printed external fixators focus on developing and assessing fixators that are personalized to a patient’s specific fracture characteristics and anatomy [9, 12, 13, 21, 22, 24, 25]. Both unilateral and circular personalized 3D printed external fixation designs have been described in the literature. The personalized fixators described in these papers were designed such that anchoring points in the fixators align with and connect to pins in the bone when the fracture is in a reduced position. Thus, fracture reduction is spontaneously achieved when the personalized 3D printed fixator is installed without needing x-rays to visualize and manipulate the fracture ends. In theory, this method of external fixation should lead to increased accuracy and precision of fracture reduction while reducing the amount of intraoperative radiation exposure, though this is yet to be verified. A direct comparison of the accuracy of 3D printed external fixators to traditional fixators has not yet been conducted, requiring further investigation. The highly customizable nature of 3D printed external fixators also allows for precise optimization of the mechanical structure to maximize strength and reduce weight, as demonstrated by the computer-based modeling by Alqahtani et al. [22] and the design by Wu et al. [9]. The computer-based modeling by Li et al. also developed a personalized fixator design that could be gradually deconstructed to adjust the stress at the fracture site as the bone heals [24].

A small number of case reports and case series conducted in China have reported successful tibial fracture management using personalized 3D printed external fixators [9, 13, 21]. All five patients treated with a 3D printed external fixator had minimal fracture displacement after initial reduction, and there were no long-term complications or loss of reduction. Spontaneous reduction was achieved on installation due to the personalized design of the fixators used in these studies. Therefore, no x-rays were needed intraoperatively during fracture reduction and fixator application. However, designing the external fixator required CT imaging of the limb in all cases, which may increase radiation exposure and costs in cases where a CT scan would not otherwise have been obtained. In addition, the operative time for installation of these 3D printed fixators was reported to be shorter than traditional external fixation for long bone fractures, with two studies [13, 21] reporting an operative time of 10 min or less to install the external fixator. While operative time may be quicker, the time from injury to definitive 3D printed external fixation application may be longer than with traditional external fixation due to the time required for designing and manufacturing the fixator. Qiao et al. reported that their fixator’s design and manufacturing process was around 20 h [13]. It is clinically recommended that debridement and fixation are performed within 24 h of injury, which may be difficult to achieve with the current manufacturing times of 3D printed fixators [28]. All three clinical studies applied a temporary external fixator to stabilize the fracture while the 3D printed external fixator was manufactured, adding to the total operative time and radiation exposure. The overall time for design and manufacturing could be reduced with the optimization of the design and manufacturing processes. It is important to note that the 3D printed external fixators successfully served as definitive treatment for all five patients studied without the need for open reduction and internal fixation. The ability to serve as a definitive treatment option is significant because patients with extensive soft tissue injury or prohibitive medical comorbidities frequently cannot undergo ORIF and require definitive treatment with external fixation.

Ultimately, further investigation, including mechanical testing, and clinical studies of 3D printed external fixation, is required to validate this technology for fracture management. Further development and validation with computational and mechanical testing is needed. Clinical trials comparing 3D printed external fixators to traditional external fixation techniques are also needed to assess outcomes and overall cost savings. In addition, variability in testing techniques and reporting of fracture displacement measures make it difficult to compare the designs currently reported in the literature. Using standardized testing methods, such as the ASTM standard already in place for mechanical testing and reporting processes would expedite and improve comparisons between studies in the future.

## Conclusions

This systematic review demonstrates that 3D printed external fixators can successfully treat complex fractures. However, the variability of designs and testing techniques currently reported on 3D printed external fixators limits the generalizability to other studies and clinical settings. While the nine papers reviewed demonstrate early successes with 3D printed external fixation devices, there is a paucity of research and data on the use of 3D printing technology in orthopaedic external fixation. Additional mechanical and clinical testing of this technology is needed.

## Data Availability

Not applicable.

## Abbreviations

3D: three dimensional
CAD: computer aided design
FEA: finite element analysis
AP: anterior posterior
ML: medial-lateral
PLA: polylactic acid
ABS: acrylonitrile butadiene styrene
CT: computed tomography
ASTM: American Society for Testing Materials
ORIF: open reduction internal fixation
